# Telephone consultations in the COVID-19 era versus pre-COVID face-to-face consultations: a survey of dermatology patient perceptions

**DOI:** 10.1007/s00403-023-02561-1

**Published:** 2023-02-16

**Authors:** Serena Ramjee, Louis Boyce, Padma Mohandas

**Affiliations:** 1 From Barts and The London School of Medicine and Dentistry, Queen Mary University of London, Garrod Building, Turner St, London, E1 2AD UK; 2 From Princess Alexandra Hospital, Harlow, UK; 3 From Whipps Cross Dermatology Department, London, UK

**Keywords:** Remote consult/consultation, Teledermatology, Telemedicine, Telehealth, COVID-19

Dear Editor, dermatologists have relied heavily on telephone consultations (TC) following the first UK national lockdown. Current UK literature comparing patient perceptions of TC to face-to-face consultations (F2FC) indicates a F2FC preference, although this research includes patients who have not experienced both F2FC and TC [3].

Between the 6th December 2021 and 16th December 2021, to assess the role of TC in secondary-care dermatology during the UK COVID-19 era, we retrospectively surveyed, via telephone, adult secondary-care dermatology patients from a single centre (London, UK) who attended one or more TC from January to February 2021 and one or more F2FC within one year before the first UK national lockdown for the same condition(s) (*n* = 157). We asked eleven questions (questions (Q)1-Q11), adapted from previous literature [1, 3]. Demographic data was acquired using patient records and Q1-Q6 (Table 1). Q7-Q11 (Fig. 1) determined patient satisfaction, consultation preference, and preference reasoning.

**Table 1:** Demographics of patients (*n* = 74) included in this study

Characteristics	*n* (%)
Age, years (*n* = 74)	
20–33	11 (14.86)
34–46	21 (28.38)
47–59	17 (22.97)
60–73	20 (27.03)
74–86	5 (6.76)
Sex (*n* = 74)	
Male	43 (58.11)
Female	31 (41.89)
Diagnostic category (*n* = 74)	
Acne	6 (8.11)
Alopecia	4 (5.41)
Eczema	11 (14.86)
Psoriasis	24 (32.43)
Other	23 (31.08)
Multiple primary diagnoses	6 (8.11)
Number of attended F2FC 1 year prior to COVID-19 (*n* = 74)	
1–2	49 (66.22)
3–4	20 (27.03)
5–6	5 (6.76)
Number of attended TC during COVID-19 (*n* = 74)	
1–2	39 (52.70)
3–4	31 (41.89)
5–6	4 (5.41)
Q1. What is your first language? (*n* = 74)	
English	59 (79.73)
Other	15 (20.27)
Q2. Do you have a disability? (*n* = 74)	
Yes	19 (25.68)
No	55 (74.32)
Q3. If yes to Q2, what is your disability? (*n* = 19)	
Autoimmune	2 (10.52)
Musculoskeletal	9 (47.37)
Neurological	2 (10.52)
Psychiatric	2 (10.52)
Multiple disabilities	4 (21.05)
Q4. Do you have hearing loss? (*n* = 74)	
Yes	6 (8.11)
No	68 (91.89)
Q5. Are you a parent of a child under 13 years or a career? (*n* = 74)	
Yes	17 (22.97)
No	57 (77.03)
Q6. How confident are you with technology? (*n* = 74)	
Very confident	23 (31.08)
Confident	39 (52.70)
Unconfident	9 (12.16)
Very unconfident	3 (4.05)

*Q* question, *TC* telephone consultations, *F2FC* face-to-face consultations

**Fig. 1:**
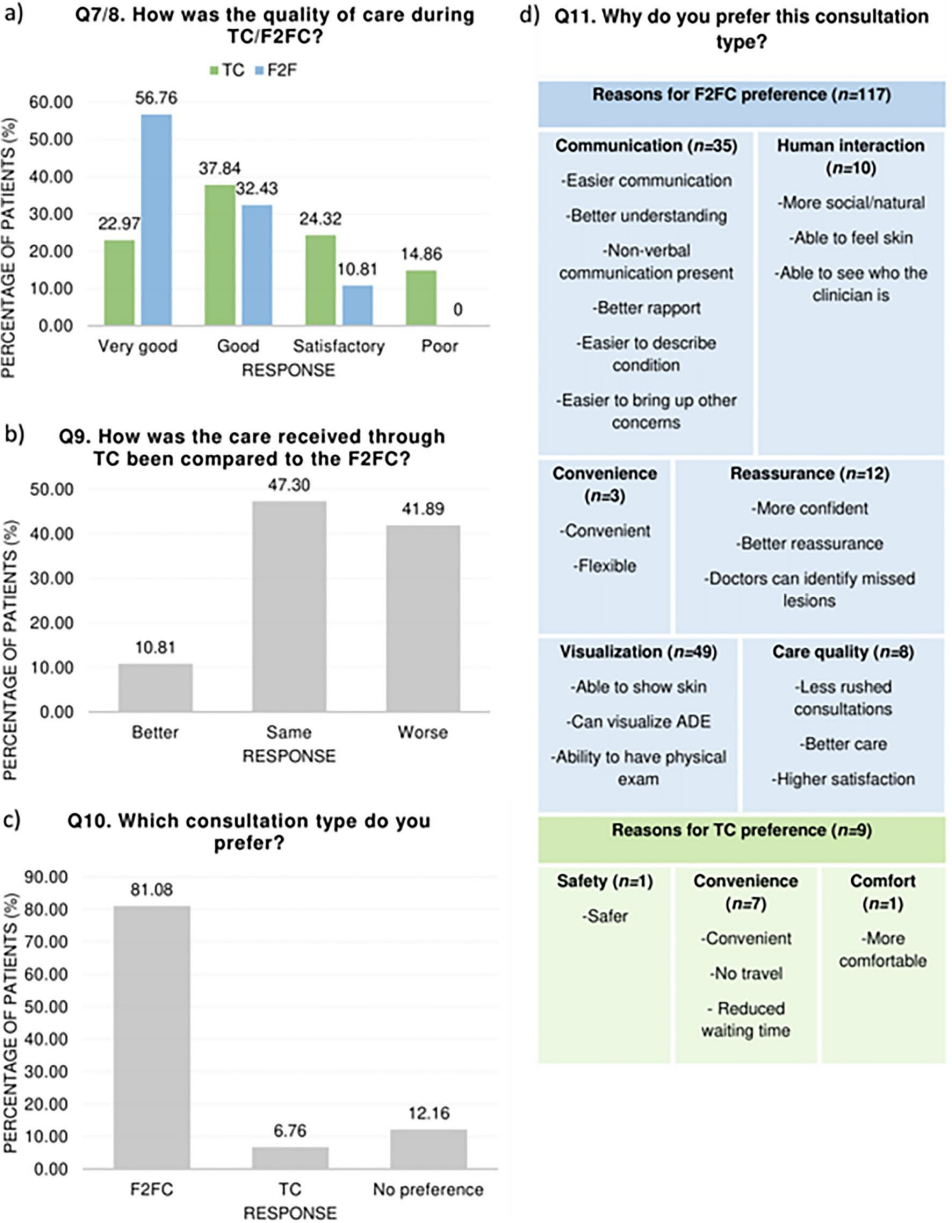
The results of satisfaction and consultation preference from four-closed ended questions, **a** Q7, Q8, **b** Q9, and **c** Q10, alongside the consultation preference reasoning themes from one open-ended question, **d** Q11. Created on Word. *Q* question, *TC* telephone consultations, *F2FC* face-to-face consultations

Seventy-four patients participated (Table 1, 47.13% response rate). Most patients were excluded because they did not answer the telephone (*n* = 34). The final cohort consisted of 43 women and 31 men with a median age of 52 years (interquartile range (IQR) = 27.25). Overall, patient satisfaction with the quality of care was significantly higher in F2FC than during TC (*p* < 0.001) (Fig. 1a), with most patients describing F2FC as “very good” (*n* = 42,56.76%) and TC as “good” (*n* = 28, 37.84%). While the majority of patients (47.30%, *n* = 35) thought that TC led to the same level of care as F2FC (Fig. 1b), 41.89% (*n* = 31) claimed that TC care was worse. Most patients (81.08%, *n* = 60) preferred F2FC (Fig. 1c). Of the 117 reasons (Fig. 1d) explaining this preference, “able to show skin” (*n* = 47) was the most common. Lack of travel (*n* = 3) was the most popular reason (*n* = 9) for preferring TC.

Our cohort’s favouritism towards F2FC likely stems from its unique advantages, including non-verbal communication. Moreover, patients may desire the normalcy of F2FC, considering we collected data following the ease of COVID restrictions and the re-introduction of in-person opportunities (March 2021). Our results support the findings of Handa et al*.* [4]*,* and Edward et al. [2], who also reported patients having an unfavourable view of TC compared to F2FC, but contrast to those documented by Gnanappiragasam et al. [3], who found no consultation preference. Most patients we hoped to recruit failed to answer the telephone, with those participating recalling almost a year back to their TC. Additionally, several patients hesitated to critique TC despite their anonymized responses. Since a degree of sample selection bias, recall bias, and social desirability bias is likely to present, future research should endeavour to collect prospective data using initial indirect surveying. Despite the F2FC preference, it is encouraging that our patients believe TC are “good”. Nevertheless, TC are an essential tool for triage and remote care, so there is a need to maximize satisfaction. Such changes are crucial for minimizing non-engagement with remote services and developing long-term care delivery strategies that adapt to COVID-19’s continuing presence, a challenge given the importance of visual inspection in dermatology [5].

## Data Availability

The datasets generated during and/or analysed during the current study are available from the corresponding author on reasonable request.
